# Parental concerns and uptake of childhood vaccines in rural Tanzania – a mixed methods study

**DOI:** 10.1186/s12889-020-09598-1

**Published:** 2020-10-20

**Authors:** Lavanya Vasudevan, Joy Noel Baumgartner, Sara Moses, Esther Ngadaya, Sayoki Godfrey Mfinanga, Jan Ostermann

**Affiliations:** 1 Department of Family Medicine and Community Health, Duke University School of Medicine, Durham, North Carolina USA; 2 Center for Health Policy and Inequalities Research, Duke University, Durham, North Carolina USA; 3 Duke Global Health Institute, Durham, North Carolina USA; 4 Muhimbili Research Centre, National Institute for Medical Research, Dar-es-Salaam, United Republic of Tanzania; 5 Muhimbili University of Health and Allied Sciences, Dar-es-Salaam, United Republic of Tanzania; 6 School of Life Sciences and Bioengineering, Nelson Mandela African Institution of Science and Technology, Arusha, United Republic of Tanzania; 7 Department of Health Services Policy & Management, Arnold School of Public Health, University of South Carolina, Columbia, SC USA; 8 South Carolina SmartState Center for Healthcare Quality, University of South Carolina, Columbia, SC USA

**Keywords:** Childhood vaccinations, Parental concerns, Vaccine hesitancy, Vaccination timeliness, Tanzania, Sub-Saharan Africa

## Abstract

**Background:**

Vaccine hesitancy has been recognized as an important barrier to timely vaccinations around the world, including in sub-Saharan Africa. In Tanzania, 1 in 4 children is not fully vaccinated. The objective of this mixed methods study was to describe and contextualize parental concerns towards vaccines in Tanzania.

**Methods:**

Between 2016 and 2017, we conducted a cross-sectional survey (*n* = 134) and four focus group discussions (FGDs, *n* = 38) with mothers of children under 2 years of age residing in Mtwara region in Southern Tanzania. The survey and FGDs assessed vaccination knowledge and concerns and barriers to timely vaccinations. Vaccination information was obtained from government-issued vaccination cards.

**Results:**

In the cross-sectional survey, 72% of mothers reported missed or delayed receipt of vaccines for their child. Although vaccine coverage was high, timeliness of vaccinations was lower and varied by vaccine. Rural mothers reported more vaccine-related concerns compared to urban mothers; literacy and access to information were identified as key drivers of the difference. Mothers participating in FGDs indicated high perceived risk of vaccine-preventable illnesses, but expressed concerns related to poor geographic accessibility, unreliability of services, and missed opportunities for vaccinations resulting from provider efforts to minimize vaccine wastage.

**Conclusions:**

Findings from our cross-sectional survey indicate the presence of vaccination delays and maternal concerns related to childhood vaccines in Tanzania. In FGDs, mothers raised issues related to convenience more often than issues related to vaccine confidence or complacency. Further research is necessary to understand how these issues may contribute to the emergence and persistence of vaccine hesitancy and to identify effective mitigation strategies.

## Background

In recent years, the coverage of routine childhood immunizations in sub-Saharan Africa (SSA) has stalled at 72% [1, 2]. Recurring vaccine-preventable outbreaks in various regions of SSA and persistent vaccine-related concerns among parents have raised worries that gains made by immunization programs in the coverage of childhood vaccines may be reversed [3–7]. In light of these concerns, there is a growing emphasis on identifying and mitigating vaccine hesitancy - the decision of parents to decline or delay vaccinations for their children [8, 9]. In the multifaceted strategy to stem vaccine-preventable outbreaks in under-immunized children, the WHO recognizes reduction of vaccine hesitancy as a key global priority [8–12].

Several drivers of vaccine hesitancy have been identified in the literature, including factors related to parental confidence in vaccines, complacency towards vaccines and vaccination programs, and the convenience of accessing vaccines [13]. These drivers may influence parental knowledge about vaccines, experiences with vaccination programs, and intention to vaccinate. Without effective interventions to mitigate these drivers of parental vaccine hesitancy, children from hesitant families will remain susceptible to vaccine-preventable diseases. However, addressing vaccine hesitancy is challenging. Parental concerns vary widely based on factors such as religious, cultural, political, geographic, or socio-economic context, vaccine type, and mode of delivery [11, 14]. Hence, the WHO suggests that, to be effective, interventions to address vaccine hesitancy be tailored to, and informed by, concerns specific to the respective target population [14, 15].

Of the countries in SSA, Tanzania, under the leadership of the National Immunization and Vaccine Development (IVD) programme, has one of the highest rates of coverage of routine childhood vaccines. Over the past several decades, the routine immunization program in Tanzania has expanded significantly to cover eleven vaccine-preventable diseases. In addition, the IVD has made substantial efforts to reduce stock outs and establish a reliable supply chain to improve vaccine availability in alignment with the Global Vaccine Action Plan’s Reach Every District (RED) goal. Despite these efforts, challenges to universal immunization remain, especially in rural areas of the country. According to the 2015–16 Demographic and Health Survey (DHS), 1 in 4 children nationally is not fully vaccinated, and many regions fall short of the 90% coverage target set by RED [16, 17]. In addition, there is significant variation in childhood vaccine coverage by region, socio-economic status, parental education, and rurality [16, 18]. To our knowledge, while many studies have examined broad barriers to vaccine uptake in Tanzania, no studies have specifically looked at parental concerns and their impact on vaccine uptake. To bridge this knowledge gap and inform the development of interventions to reduce vaccine hesitancy and improve uptake of basic childhood vaccinations, we used a mixed methods approach to describe and contextualize parental concerns towards vaccines in southern Tanzania.

## Methods

### Study setting

The methods described in this study were part of a larger study that aimed to understand barriers to timely vaccine uptake in southern Tanzania and develop a digital health intervention to promote timely vaccine uptake. The study was conducted in one urban district (Mtwara Municipality) and one rural district (Mtwara District Council) in Mtwara Region in Southeastern Tanzania. Mtwara Region has an estimated population of 1.3 million people; the two districts included in this study have an estimated population of 336,000 [19, 20]. The Tanzania Ministry of Health, Community Development, Gender, Elderly and Children and the national Immunization and Vaccine Development (IVD) program oversee the provision of routine childhood vaccinations in Mtwara Region. This study was limited to government health facilities which provide the vast majority of childhood vaccinations. In 2015–16, coverage of all basic childhood vaccines as per national guidelines (Supplementary Table 1) in Mtwara Region was estimated to be 79%, mirroring the national coverage rate of 75% [16].

### Cross-sectional survey

The methods of the cross-sectional survey are reported below in accordance with the STROBE checklist for cross-sectional studies (Supplementary Table 2).

#### Study participants

Women ages 16 years or older, with children ages 12–23 months, were eligible to participate in the cross-sectional survey on vaccination knowledge, concerns, and practices. The minimum child age for the survey was set to 12 months to allow for the assessment of vaccine uptake in their first year of life.

#### Sample size

A priori power calculations suggested that a stratified sample of 10–12 women per facility from 12 health facilities would yield adequate statistical power (> 0.8) to characterize differences in sociodemographic characteristics between rural and urban mothers and to identify medium to strong correlates of vaccine hesitancy.

#### Recruitment

Between May and June 2017, we used a combination of purposive and snowball sampling strategies to recruit mothers from 4 urban and 8 rural government health facilities and the surrounding communities for participation in the cross-sectional survey. Trained research assistants approached eligible women presenting with children to the well child clinic at participating facilities for consent and enrollment. To reduce biases from facility-based enrollment, participating women were asked to identify other potentially eligible women in their communities. Trained research assistants approached referred women in their homes for eligibility determination, consent, and enrollment.

#### Data collection

Trained research assistants conducted cross-sectional surveys with mothers at health facilities, homes, or other mutually agreed-upon locations. The survey was interviewer administered, and data were collected on a tablet device using the Qualtrics^XM^ survey platform. Vaccination knowledge and concerns were assessed using questions adapted from a WHO survey on vaccine hesitancy [21], and a prior study (see Acknowledgements). Other survey questions assessed women’s sociodemographic characteristics, reproductive history, and barriers to vaccinations. Children’s vaccination histories in their first year of life, including dates of vaccinations, were obtained from government-issued vaccination cards.

#### Key outcomes

The key outcomes of interest, mothers’ vaccine hesitancy and the timeliness of children’s vaccinations, were measured as follows:

- *Vaccine hesitancy:* For each of 15 survey questions used to assess vaccine hesitancy, the mother’s response was scored as 0 if not hesitant, 1 if not sure, or 2 if hesitant (survey items were reverse coded as needed). A vaccine hesitancy index score was generated for each mother as the sum of the individual item scores. The index ranged from 0, if the mother scored not hesitant for all items, to 30, if the mother scored hesitant on all items.
- *Timeliness of vaccinations:* For each vaccine received by the child, the date of vaccination was coded with a score of 1 if timely or 0 if early, late, or missed. Vaccinations received prior to the due date were considered early; those received ≥28 days beyond the due date were considered late (see Supplementary Table 3). Timeliness of individual doses in a vaccine series was calculated contingent on the date of receipt of the previous dose. Thus, a child could be timely for the receipt of the second or third dose of a vaccine even if the previous dose was delayed.

#### Data analysis

Survey data were analyzed using Stata v.15 (StataCorp LLC). Distributions of the key outcomes of interest, sociodemographic characteristics of mothers, and other correlates of vaccine hesitancy and vaccination decisions, were described using means and ranges for continuous variables and proportions for categorical variables. Variation in these characteristics between rural and urban mothers were analyzed using Student’s *t*-tests and chi-squared tests for continuous and categorical variables, respectively. Associations between sociodemographic characteristics, vaccine hesitancy, and vaccine coverage and timeliness were assessed using linear least squares regression models that accounted for clustering at the level of the referral, i.e., each index woman enrolled from a health facility and her community-based referrals formed a cluster. Observations with missing data were excluded from the respective analyses.

### Focus group discussions

#### Study participants

Women ages 16 years or older, with children ages 0–23 months, were eligible to participate in focus group discussions (FGDs) on barriers to childhood vaccination.

#### Sample size

As is typical of qualitative analyses, the goal was to identify community norms and common themes across groups. Given broad eligibility criteria we did not anticipate significant differences between participants across groups; groups were expected to be similar except for their geographic location. Due to these considerations and published reports on qualitative sample size considerations [22–24], we determined a priori that four FGDs with 10 women per FGD would likely be sufficient to reach saturation.

#### Recruitment

Between December 2016 and February 2017, a purposive sampling approach was used to recruit mothers from two urban and two rural government health facilities for participation in FGDs. Trained research assistants approached eligible women for consent and enrollment.

#### Data collection

Two female Tanzanian research assistants (RAs) trained in qualitative data collection conducted four FGDs with mothers using a semi-structured guide aimed at understanding locally and socio-culturally relevant barriers to timely vaccinations. FGD domains consisted of open-ended questions with probes on the role of women in vaccination decision-making, and barriers to vaccine uptake. Each FGD included approximately 10 participants and lasted between 40 and 60 min. FGDs were audio-recorded, transcribed in Swahili, and translated into English for analysis. A short survey captured basic demographic information of FGD participants; field notes were written after the FGDs by the RAs. Owing to logistical considerations and variations in English literacy, transcripts were not returned to participants for comments.

#### Data analysis

Translated FGD transcripts were uploaded into QSR NVivo v.11, and thematic analyses were conducted utilizing four interrelated steps: reading, coding, data display, and data reduction. Within FGDs, participants organically discussed issues related to vaccine hesitancy. To summarize and synthesize those discussions, transcripts were coded by the first author using a codebook made of a priori, structural codes based on the “3 Cs” model of vaccine hesitancy, comprising convenience, complacency, and confidence, with an eye towards identifying community norms on these issues (see Supplementary Table 4) [25]. Narrative summaries were created for each of the “3 Cs” domains and are presented below with accompanying quotes from the mothers for illustration. The narrative summaries were routinely shared with the study team and feedback was incorporated.

## Results

### Cross-sectional survey

Table 1 shows the socio-demographic characteristics of the women participating in the cross-sectional survey (*n* = 134). The sample included 45 index clients enrolled from health facilities, and 89 referrals enrolled from the surrounding communities. There were no statistically significant differences between rural vs. urban women in the study with respect to the distributions of their ages, marital status, or employment status. However, more rural women than urban women reported a lack of formal education (34.8% vs. 11.9%) and were unable to read and write a whole sentence (50% vs. 14.3%). While urban women reported more household assets, rural women reported greater media exposure (defined as watching television almost every day). Compared to urban women, rural women also reported traveling greater distances (walking time) to the vaccination clinic.

**Table 1:** Sociodemographic characteristics of women participating in the cross-sectional survey (*N* = 134)

Characteristic	Response categories	All participants N or mean % or (sd)		Urban participants N or mean % or (sd)		Rural participants N or mean % or (sd)		Significance
		*N* = 134		*N* = 42		*N* = 92		
**Age**	(in years)	26.5	(6.9)	25.0	(5.0)	27.2	(7.6)	
**Education**	None	37	27.6	5	11.9	32	34.8	***
	Some primary school	12	9.0	1	2.4	11	12.0	
	Primary school completed	58	43.3	19	45.2	39	42.4	
	Secondary school	27	20.1	17	40.5	10	10.9	
**Marital status**	Married	84	62.7	29	69.0	55	59.8	ns
	Divorced / separated / widowed	30	22.4	6	14.3	24	26.1	
	Never married	20	14.9	7	16.7	13	14.1	
**Parity**	Any prior children	77	57.5	19	45.2	58	63.0	ns
	No prior children	57	42.5	23	54.8	34	37.0	
**Literacy**	Unable to read and write whole sentence	52	38.8	6	14.3	46	50.0	***
	Able to read and write whole sentence	82	61.2	36	85.7	46	50.0	
**Media exposure**	Watches television almost every day	103	76.9	19	45.2	84	91.3	***
	Watches television weekly or less	31	23.1	23	54.8	8	8.7	
**Household assets**	Number of assets (0-10)	2.7	(2.5)	4.7	(2.7)	1.8	(1.8)	***
**Walking time to dispensary**	<15 minutes	81	60.4	32	76.2	49	53.3	**
	15-29 minutes	27	20.1	8	19.0	19	20.7	
	30+ minutes	26	19.4	2	4.8	24	26.1	

Notes: ***p < 0.001, **p < 0.01. *p < 0.05, *ns* not significant

**Fig. 1:**
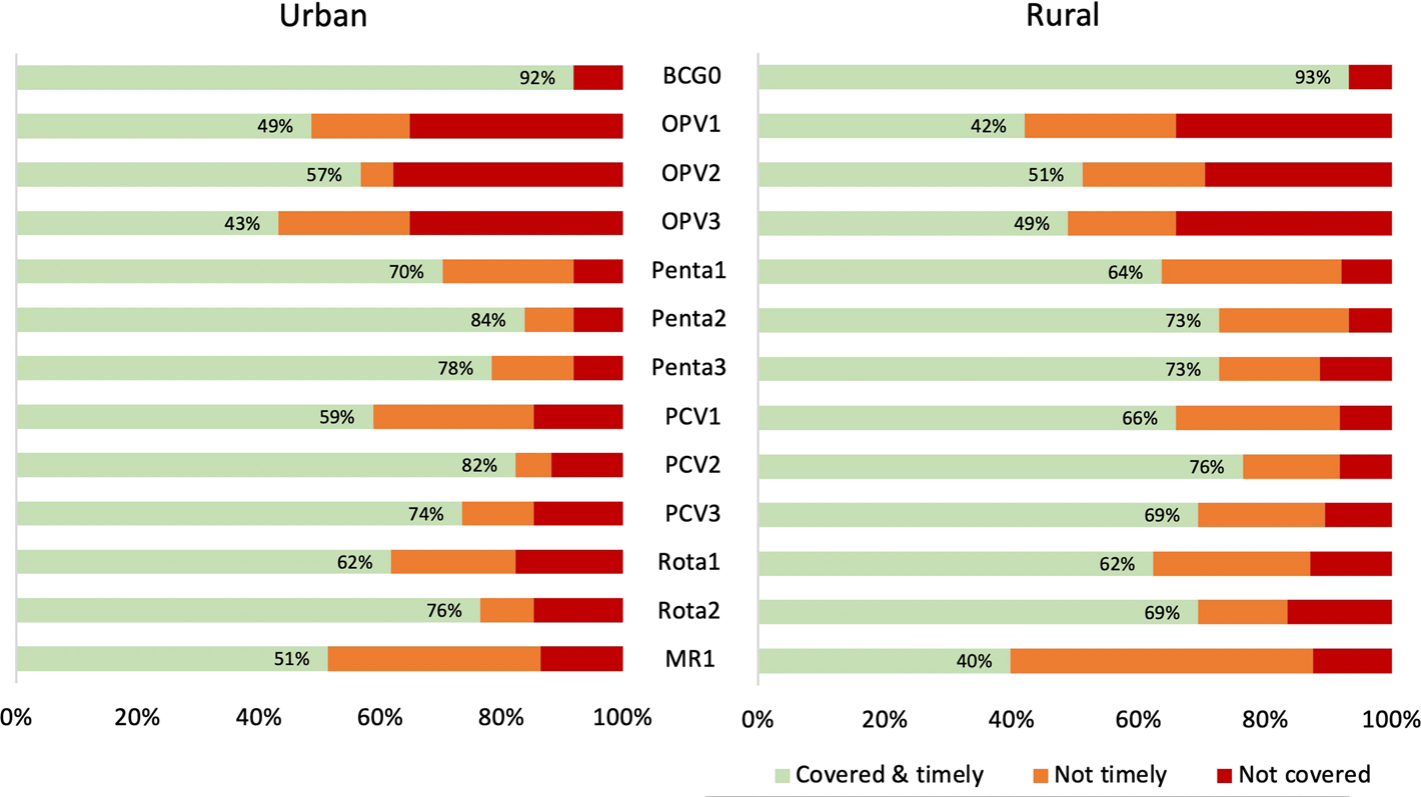
Childhood vaccination coverage and timeliness for urban and rural children for vaccinations due before 12 months of age (*n* = 125). Notes: Percentages are based on vaccination dates reported on government-issued vaccination cards for 37 urban and 88 rural children 12–23 months of age. See Supplemental Table 1 for abbreviations of vaccinations. PCV* and Rota* percentages are based on data for 34 urban and 85 rural children. Nine children were excluded due to missing vaccination cards

Figure 1 and Supplementary Figure 1 show the distribution of children’s vaccination coverage and timeliness by 12 months of age. Although vaccine coverage and timeliness were similar between rural and urban settings, rates varied widely by vaccine. Vaccination coverage was lowest for the oral polio vaccine (OPV1 and OPV3, 65.6%; OPV2, 68%; numbers not shown), and highest for the birth dose of the Bacillus Calmette Guerin vaccine (BCG0, 92.8%) and the second dose of the pentavalent vaccine (Penta2, 92.8%). With the exception of BCG0, timeliness of vaccinations was low for all vaccines, with the largest delays reported in the first dose of the Measles-Rubella vaccine (MR1, 43.2% timely).

A majority of the mothers (72%, *n* = 96) participating in the survey self-reported that their child did not receive vaccine(s) they were supposed to get (results not shown). Primary reasons reported by mothers for missed vaccinations were the unavailability of vaccines (*n* = 42, 44%) and being asked to return at another time by clinic staff (*n* = 35, 36%). Other reasons included maternal or child sickness, travel, and forgetfulness. Reasons related to complacency or vaccine confidence were mentioned, but with very low frequencies. A few women thought vaccines were not needed (*n* = 4), not effective (*n* = 1), had a bad experience with previous vaccination (*n* = 1), or had a family member or friend who advised against it (*n* = 1).

**Fig. 2:**
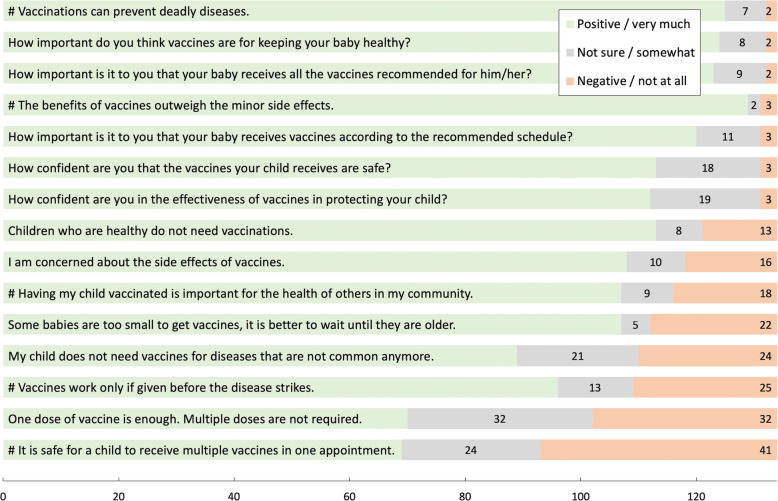
Distribution of maternal knowledge, attitudes, and concerns about childhood vaccines (*N* = 134). Notes: Numbers indicate counts of women with negative (red) or ambivalent (grey) responses to vaccination-related knowledge and attitude questions. Confidence and importance items were assessed on a 3-point scale including ‘very much’, ‘somewhat’, and ‘not at all’. Knowledge and attitude items were assessed on a 3-point scale including ‘agree’, ‘not sure’, and ‘disagree’. # indicates positively-framed items; all other items were reverse-coded

**Fig. 3:**
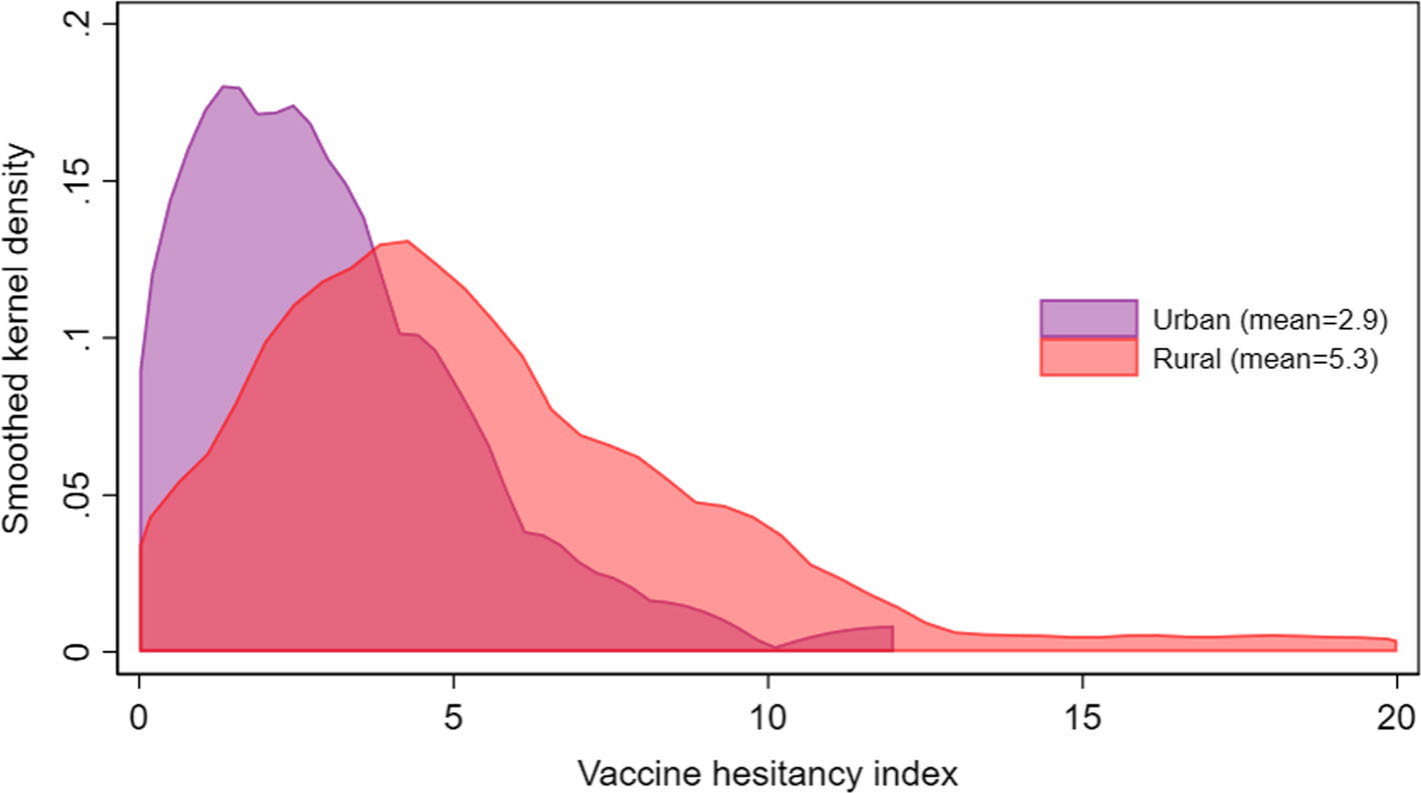
Distribution of vaccine hesitancy in rural vs. urban areas (*N* = 134). Notes: Vaccine hesitancy index defined using mothers’ responses to 15 questions assessing maternal knowledge, attitudes, and concerns about childhood vaccines; each question is scored on a scale of 0–2; see Fig. 2. A higher index indicates greater vaccine hesitancy

Figure 2 shows the distribution of mothers’ concerns and knowledge related to vaccines. While most women (93%, *n* = 125) correctly reported knowing that vaccines can prevent deadly diseases, more than a quarter (28.4%, *n* = 38) did not know that vaccines were only effective when given before a child is ill. Nearly as many mothers (23.9%, *n* = 32) did not know that multiple doses of a vaccine may be required before full immunization is achieved. The top concern (48.5%, *n* = 65) was related to the receipt of multiple vaccines in a single appointment. Overall, 79% of mothers (*n* = 99) reported at least one vaccine-related concern, and 32% of mothers (*n* = 43) reported three or more vaccine-related concerns. On average, urban women had a lower vaccine hesitancy score compared to rural women (Fig. 3).

Table 2 shows the results of four models of vaccine hesitancy. Model 1 describes the mean difference in hesitancy between rural and urban mothers: rural residence was associated with significantly higher vaccine hesitancy (*p* < 0.001). Model 2 (“Information model”) adds variables indicative of access to information (which is conceptually related to the confidence and complacency domains of the 3 C’s model): literacy (*p* = 0.033) and media exposure (*p* = 0.024), but not formal education, were negatively associated with vaccine hesitancy. In Model 3 (“Access model”), variables related to physical access and household economic wellbeing (which are related to the convenience domain of the 3 C’s model) were not associated with vaccine hesitancy. Model 4 includes the full set of covariates; only literacy (*p* = 0.041) and media exposure (*p* = 0.037) were significantly associated with vaccine hesitancy. In Models 2–4, after controlling for demographic, information, access, and/or other characteristics, the difference in vaccine hesitancy between rural and urban mothers was no longer statistically significant at conventional levels (range *p* = 0.057 to *p* = 0.109).

**Table 2:** Correlates of vaccine hesitancy among women participating in the cross-sectional survey (*N* = 134)

Characteristic	Response category	Rural vs. urban	Information model	Access model	Full model
**Place of residence**	Rural (vs. urban)	2.44*** (0.54)	1.16 (0.71)	1.79 (0.85)	1.45 (0.74)
**Mother's age**	(in years)				-0.07 (0.07)
**Education**	Some primary school (vs. no schooling)		-0.47 (1.04)		-1.10 (1.37)
	Primary school completed		1.35 (1.39)		1.00 (1.73)
	Secondary school		0.91 (1.54)		0.43 (1.87)
**Marital status**	Divorced / separated / widowed				-0.25 (0.76)
	Never married				-0.08 (0.88)
**Parity**	First child (vs. prior children)				-0.01 (0.84)
**Literacy**	Able to read and write whole sentence (vs. unable)		-2.61* (1.19)		-2.66* (1.26)
**Media exposure**	Watches TV almost every day (vs. weekly or less)		-1.53* (0.65)		-2.39* (1.12)
**Household assets**	# of assets in household (0-10)			-0.19 (0.14)	0.26 (0.26)
**Distance to nearest dispensary**	15-29 minutes walking (vs. <15 min)			1.02 (0.82)	0.91 (0.79)
	30+ minutes walking			0.43 (0.65)	0.52 (1.04)
**Constant**		2.90*** (0.36)	5.01*** (0.89)	3.57*** (0.80)	6.26** (2.27)
**Number of observations**		134	134	134	134
**R-squared**		0.10	0.18	0.13	0.21
**AIC**		712.9	709.7	714.8	718.9

Notes: Estimates from linear least squares regression models. Robust standard errors in parentheses

*** *p* < 0.001, ** *p* < 0.01, * *p* < 0.05

*Abbreviations*: *min* Minutes, *AIC* Akaike information criterion

### Focus group discussions

In total, 38 mothers participated in the FGDs. Mothers in the FGDs were between 17 and 37 years of age. While all women reported that their youngest child received vaccines, eight women (21%) women reported vaccination delays and 2 women (5%) reported missed vaccinations.

All “3 C’s” (convenience, confidence and complacency) were discussed by the women in the FGDs. Concerns predominantly centered on the convenience of accessing vaccines, followed by issues related to vaccine confidence. Only a few comments pertained to complacency, and were usually based on experiences of FGD participants in community settings and directed at “other” women. Comments related to satisfaction with the vaccination experience and trust in provider recommendations and actions were mentioned in the context of convenience, confidence, and complacency.

#### “3 Cs” of vaccine hesitancy*: Convenience*

Women in the FGDs highlighted a number of challenges related to the convenience of getting a child vaccinated. These included poor geographic accessibility of facilities and lack of affordable transportation options, especially for women residing in rural areas.

*“Some of us come very far from here, so we have a problem of transport. Sometimes the date for vaccination may reach but I miss transport to go to the hospital.”*

*Rural Mother*

In general, women expressed frustration when experiencing delays beyond their control, primarily due to unreliability of supplies needed for vaccination at the health facilities to keep refrigerators functioning.

*“We do not miss completely but vaccinations are sometimes delayed because of shortage of supplies.”*

*Rural Mother*

Women also expressed displeasure at being turned away when, in an effort to minimize vaccine wastage, providers were reluctant to open multidose vials for the small number of children present to be vaccinated.

*“I think the idea of telling us to come back another day because we are only two or three should end because we have left our activities to come on that day and others who didn’t come maybe they are sick at home so how long should we wait for them, I think that habit should stop and we should get the vaccine no matter how many we are.”*

*Urban Mother*

#### “3 Cs” of vaccine hesitancy: Complacency

Comments relating to complacency described varying knowledge about vaccines, motivation to vaccinate, and household decision-making and support for vaccinations. Similar to the study’s quantitative findings, women in the FGDs demonstrated high perceived risk of vaccine-preventable illnesses, and generally agreed that vaccines were important for their child’s health. When probed about household decision-making related to the child’s vaccinations, most women suggested either having autonomy or being able to make shared decisions with their spouses. Women reported varied roles for the father of the child in vaccination decision-making and uptake. These roles ranged from escorting the mother and child to the health facility, indifference to what health services the child may have received, to scolding mothers for the occurrence of post-vaccination fever in children. One mother talked about the support she received from her husband as follows:

*“…I thank God because when it comes to his children, my husband provides a lot of care and with regards to vaccinations, when I forget he usually reminds me like today. He is the one who told me the day before yesterday that today was the day for vaccination that I should bring the child and as we speak I came along with him so he is just outside”*

*Rural Mother*

Another mother articulated the household dynamics of vaccination decision-making as follows:

*“The mother is the one who makes all the decisions concerning the child, because if you tell him the vaccination date, other men start quarrelling and it can reach separation: ‘I don’t want my child to be injected, you will give her fever, you will give me problems and later I will take her to the hospital again’, so you have to say ‘No’, that it protects the child from other things, so you have to make it a priority to take a child to the clinic to get the shots”*

*Urban Mother*

Some women in the focus groups referred to others in the community who lacked knowledge about vaccines or knowledge of its importance, and suggested a role for health providers in educating them.

*“In short they think that after getting pregnant and giving birth safely, they just leave without caring, they don’t care whether the children are vaccinated or not, so long as they have given birth then they think that the child will just grow so they don’t care about vaccinating the child.”*

*Rural Mother*

#### “3 Cs” of vaccine hesitancy*: Confidence*

Many women in the FGDs expressed concerns related to the discomfort and minor side effects of vaccinations in children (e.g., crying, and formation of abscesses). In response to a question on multiple vaccinations administered during one visit, one woman said her decision to vaccinate her child had changed because of multiple injections, while others said their decision remained the same. In general, women reported that they relied on the doctor or nurse to tell them about which vaccines are due and when they are scheduled.

*“We don’t make choices, it is the nurse who tells us the date and place to take our babies for vaccination. We are always told that when we go for clinic, the nurse looks at the card and tells me a date I am supposed to bring my baby for vaccination. That day I keep in mind”*

*Rural Mother*

## Discussion

To our knowledge, this study is the first to assess vaccine hesitancy and parental concerns and their association with vaccine uptake in Tanzania. Among 134 rural and urban children in Southern Tanzania, vaccination coverage was high, but vaccination timeliness was low. Both quantitative survey data from the mothers of these children, and qualitative data from FGDs suggest delays in vaccinations, and that convenience, confidence, and to a lesser degree, complacency, may contribute to these delays. Two previous studies in the same area described low timeliness of vaccine uptake in the region but did not examine parental concerns [26, 27].

According to the 2015–16 Tanzania DHS, there are stark differences in the uptake of childhood vaccines in urban and rural settings [16]. These disparities are highlighted in our study in the varying number and nature of concerns among urban and rural women. Results from our cross-sectional survey suggest that access to information may be a key driver of differences between rural and urban areas. Based on a study of community vaccine perceptions, Chambongo et al. suggested health promotion, community sensitization, and improving provider-patient relationships, as strategies for increasing vaccination coverage among rural Tanzanian children [28]. Similar recommendations on the need to improve caregiver knowledge about vaccines were published by Magodi et al. [27].

Much of the literature on vaccine hesitancy focuses on high-income countries (HICs), where the prevalence of parental concerns towards vaccines and the link between parental concerns and sub-optimal vaccine uptake are well established [10, 29]. In contrast to HICs, data from low- and middle-income countries (LMICs) on parental concerns and their impact on vaccine uptake are lagging [30]. While parental concerns towards vaccines in HICs focus on risk-benefit tradeoffs of vaccines and issues like autism [31], concerns in LMICs tend to focus on a lack of information about vaccines and the benefits of vaccinations, and a lack of access to vaccines [13]. Much of the focus in Tanzania and other LMICs remains on improving the geographic coverage of vaccination programs. While increasing exposure to vaccines has resulted in greater awareness and dialog around vaccines in Tanzania, there may be insufficient emphasis on studying and mitigating the emergence or persistence of parental concerns.

An important finding of our study is that higher literacy and media exposure are associated with lower vaccine hesitancy among study participants. The impact of literacy is consistent with other published reports [32, 33]. Exposure to negative media content, however, has been associated with higher vaccine hesitancy in other HICs and LMICs [34–36]. In our study, we did not assess sentiments associated with specific media content related to vaccines. Further research is needed to explain the detailed relationships between media exposure relevant to vaccinations, sentiments, and vaccine hesitancy in Tanzania.

In sub-Saharan Africa, additional challenges have been noted in the form of infrequent health system contacts and missed opportunities for vaccination [4, 6, 27, 37–39]. The WHO definition of vaccine hesitancy focuses on the choice of parents to delay or refuse vaccines when vaccines are available as opposed to non-vaccination due to lack of services or interruptions to the vaccine supply or cold-chain. In our focus groups, mothers expressed frustration when turned away from health facilities despite their interest in vaccinations, either because there were insufficient numbers of children for vaccination that day, or due to lack of electricity impacting operation of the vaccine refrigerators. Policies for minimizing vaccine wastage may contribute to missed opportunities for vaccinations in these settings, particularly in rural areas with lower-volume health facilities and greater geographic access barriers for mothers. We posit that service unavailability or refusal could contribute to reduced trust, perceived unreliability of the health system, and decreased convenience of accessing vaccines. Further research is necessary to understand how lack of service availability, low reliability, and inconvenience may contribute to the emergence of vaccine hesitancy in otherwise non-hesitant families.

The focus group participants in this study agreed on the key role of mothers in healthcare decisions related to their child, while also describing various degrees of involvement by fathers. Male involvement in maternal and child health services has been shown to improve uptake of preventive health behaviors [40]. In the context of vaccinations, male involvement may help reduce delays in instances where the mother is sick, traveling, or otherwise unable to bring the child to the health facility. Further investigations of paternal perceptions towards vaccines and factors influencing their participation in child health services are important to understand the household dynamics surrounding vaccination decision-making and access of vaccination services.

Study limitations include the small sample size and the reliance on facility-based recruitment (sampling bias); in addition, the validity of the vaccine hesitancy measure in this context is unknown. There are currently few validated surveys to measure vaccine hesitancy in LMICs [41, 42]. Currently available scales for measuring vaccine hesitancy such as the Vaccine Hesitancy Scale [31] and the Parent Attitude about Childhood Vaccines (PACV) [21, 43, 44] are validated among parents from HICs. Recently, Wallace et al. presented findings from the development of a scale to measure parental vaccine acceptance in Ghana, but this scale is yet to be tested in other sub-Saharan Africa settings for its reliability and validity in measuring vaccine hesitancy [45]. Within these limitations, our study did not identify a statistically significant link between parental concerns and vaccine uptake or timeliness in Southern Tanzania; a larger sample size, a representative community-based sample, and locally validated measures of hesitancy may be needed to derive conclusive evidence about the link between parental concerns and vaccine uptake and timeliness. Finally, FGDs were designed to capture barriers to timely vaccine uptake broadly, and discussions were not tailored specifically to capture vaccine hesitancy.

## Conclusion

The findings of this study support existing literature on low timely vaccine coverage in children from Southeastern Tanzania [26, 27] and describe an underlying, unaddressed, current of vaccine hesitancy. The study highlights missed opportunities for routine immunization, common parental concerns, and the potential role of information access, which, if addressed, may improve childhood vaccination rates, especially in rural areas. Further studies are needed to examine reasons for higher vaccine hesitancy among rural mothers and develop interventions to reduce these concerns. Tailored behavior change strategies for implementation within routine immunization services may hold potential to improve the timeliness of childhood vaccinations in Tanzania. The study findings were shared with representatives of the national Immunization and Vaccine Development program and were well received. A community health worker-delivered intervention for improving vaccination knowledge and beliefs is currently being piloted in Mtwara Region [46].

## Supplementary information

**Additional file 1: Table S1.** Routine childhood vaccination schedule in Tanzania. **Table S2.** STROBE Statement. **Table S3.** Timeliness windows by dose of vaccine. **Table S4.** The “3 Cs” model for vaccine hesitancy. **Figure S1.** Sankey diagram describing vaccination coverage and conditional timeliness among 134 children in their first year of life. **Cross-sectional survey questionnaire. Focus group discussion guide**.

## Data Availability

The data that support the findings of this study are available from the National Institute for Medical Research, Muhimbili Research Centre, Tanzania, but restrictions apply to the availability of these data, which were used under a data transfer agreement for the current study, and thus are not publicly available. Data may be made available by the authors upon reasonable request and with permission of the National Medical Research Review Committee at the National Institute for Medical Research (NIMR) in Tanzania.

## Abbreviations

BCG: Bacillus Calmette Guerin
DHS: Demographic and Health Survey
DPT: Diphtheria-Pertussis-Tetanus
FGD: Focus Group Discussion
HIC: High Income Country
IRB: Institutional Review Board
IVD: Immunizations and Vaccine Development
LMICs: Low- and middle-income countries
MACH: Maternal, Adolescent, and Child Health
MCV: Measles containing vaccine
MR: Measles Rubella
OPV(x): Oral Polio Vaccine; x indicates the first, second, or third dose in vaccine series. Zero indicates dose due at birth
PCV(x): Pneumococcal vaccine; x indicates the first, second, or third dose in the vaccine series
Penta(x): Pentavalent vaccine (DPT-Haemophilus Influenzae B-Hepatitis B); x indicates the first, second, or third dose in the vaccine series
Rota(x): Vaccine for rotavirus infection; x indicates the first or second dose in the vaccine series
VHI: Vaccine Hesitancy Index
WHO: World Health Organization
