# Supplementary material for: Parental concerns and uptake of childhood vaccines in rural Tanzania – a mixed methods study
Source: BMC Public Health. 2020 Oct 20;20:1573. doi: 10.1186/s12889-020-09598-1 (PMC7573867; doi:10.1186/s12889-020-09598-1)
Supplement: Supplementary file 1 — Additional file 1: Table S1. Routine childhood vaccination schedule in Tanzania. Table S2. STROBE Statement. Table S3. Timeliness windows by dose of vaccine. Table S4. The “3 Cs” model for vaccine hesitancy. Figure S1. Sankey diagram describing vaccination coverage and conditional timeliness among 134 children in their first year of life. Cross-sectional survey questionnaire. Focus group discussion guide. [file 12889_2020_9598_MOESM1_ESM.docx]

**SUPPLEMENTARY FILES**

**Supplementary Table 1: Routine childhood vaccination schedule in Tanzania**

| **Antigen** | **Schedule** |
| --- | --- |
| BCG, OPV0 | At birth or first contact |
| OPV1, Penta1, PCV1, Rota1 | 6 weeks of age |
| OPV2, Penta2, PCV2, Rota2 | 10 weeks of age |
| OPV3, Penta3, PCV3, IPV | 14 weeks of age |
| MR1 | 9 months of age |
| MR2 | 18 months of age |

*Abbreviations:* BCGx, Bacillus Calmette Guerin; OPVx, Oral Polio Vaccine; Pentax, Pentavalent vaccine comprising antigens of diphtheria, pertussis, tetanus, Haemophillus influenza B, and hepatitis B; PCVx, Pneumococcal vaccine; Rotax, Rotavirus vaccine; IPVx, Injectable Polio Vaccine; MRx, Measles and Rubella vaccine; x refers to the dose number in the vaccine series.

**Supplementary Table 2: STROBE Statement**

|  | Item No | Recommendation | Page No |
| --- | --- | --- | --- |
| **Title and abstract** | 1 | (*a*) Indicate the study’s design with a commonly used term in the title or the abstract | 1 |
|  |  | (*b*) Provide in the abstract an informative and balanced summary of what was done and what was found | 2-3 |
| Introduction | | | |
| Background/rationale | 2 | Explain the scientific background and rationale for the investigation being reported | 7-8 |
| Objectives | 3 | State specific objectives, including any prespecified hypotheses | 8 |
| Methods | | | |
| Study design | 4 | Present key elements of study design early in the paper | 8 |
| Setting | 5 | Describe the setting, locations, and relevant dates, including periods of recruitment, exposure, follow-up, and data collection | 9 |
| Participants | 6 | (*a*) Give the eligibility criteria, and the sources and methods of selection of participants | 9, 12 |
| Variables | 7 | Clearly define all outcomes, exposures, predictors, potential confounders, and effect modifiers. Give diagnostic criteria, if applicable | 11 |
| Data sources/ measurement | 8 | For each variable of interest, give sources of data and details of methods of assessment (measurement). Describe comparability of assessment methods if there is more than one group | 10, 12 |
| Bias | 9 | Describe any efforts to address potential sources of bias | 10 |
| Study size | 10 | Explain how the study size was arrived at | 10, 12 |
| Quantitative variables | 11 | Explain how quantitative variables were handled in the analyses. If applicable, describe which groupings were chosen and why | 11 |
| Statistical methods | 12 | (*a*) Describe all statistical methods, including those used to control for confounding | 11 |
|  |  | (*b*) Describe any methods used to examine subgroups and interactions | Not applicable |
|  |  | (*c*) Explain how missing data were addressed | 11 |
|  |  | (*d*) If applicable, describe analytical methods taking account of sampling strategy | Not applicable |
|  |  | (*e*) Describe any sensitivity analyses | Not applicable |
| Results | | | |
| Participants | 13 | (a) Report numbers of individuals at each stage of study—eg numbers potentially eligible, examined for eligibility, confirmed eligible, included in the study, completing follow-up, and analysed | Not applicable |
|  |  | (b) Give reasons for non-participation at each stage | Not applicable |
|  |  | (c) Consider use of a flow diagram | Not applicable |
| Descriptive data | 14 | (a) Give characteristics of study participants (eg demographic, clinical, social) and information on exposures and potential confounders | 13, Table 1 |
|  |  | (b) Indicate number of participants with missing data for each variable of interest | Figure 1 |
| Outcome data | 15 | Report numbers of outcome events or summary measures | Figures 1-3, Table 1, 2 |
| Main results | 16 | (*a*) Give unadjusted estimates and, if applicable, confounder-adjusted estimates and their precision (eg, 95% confidence interval). Make clear which confounders were adjusted for and why they were included | Table 2 |
|  |  | (*b*) Report category boundaries when continuous variables were categorized | Table 1, Table 2 |
|  |  | (*c*) If relevant, consider translating estimates of relative risk into absolute risk for a meaningful time period | Not applicable |
| Other analyses | 17 | Report other analyses done—eg analyses of subgroups and interactions, and sensitivity analyses | Table 1, Table 2 |
| Discussion | | | |
| Key results | 18 | Summarise key results with reference to study objectives | 19 |
| Limitations | 19 | Discuss limitations of the study, taking into account sources of potential bias or imprecision. Discuss both direction and magnitude of any potential bias | 21 |
| Interpretation | 20 | Give a cautious overall interpretation of results considering objectives, limitations, multiplicity of analyses, results from similar studies, and other relevant evidence | 19-22 |
| Generalisability | 21 | Discuss the generalisability (external validity) of the study results | 21 |
| Other information | | | |
| Funding | 22 | Give the source of funding and the role of the funders for the present study and, if applicable, for the original study on which the present article is based | 5 |

**Supplementary Table 3: Timeliness windows by dose of vaccine**

| **Vaccine** | **Recommended window** | **Considered timely if administered…** |
| --- | --- | --- |
| BCG0 | At birth or first contact | Prior to or same as Penta1 date |
| OPV0 | At birth or first contact | Prior to or same as Penta1 date |
| OPV1, Penta1, PCV1, Rota1 | 6 weeks of age | 42-70 days post-birth |
| OPV2, Penta2, PCV2, Rota2 | 4 weeks after dose 1 | 28-56 days post OPV1, Penta1, PCV1, Rota1, respectively |
| OPV3, Penta3, PCV3, IPV | 4 weeks after dose 2 | 28-56 days post OPV2, Penta2, PCV2, Rota2, respectively |
| MR1 | 9 months of age | 266-294 days post-birth |

*Abbreviations:* BCGx, Bacillus Calmette Guerin; OPVx, Oral Polio Vaccine; Pentax, Pentavalent vaccine comprising antigens of diphtheria, pertussis, tetanus, Haemophillus influenza B, and hepatitis B; PCVx, Pneumococcal vaccine; Rota, Rotavirus vaccine; IPVx, Injectable Polio Vaccine; MRx, Measles and Rubella vaccine; x refers to the dose number in the vaccine series (x = 0, 1, 2 or 3, depending on the vaccine).

**Supplementary Table 4: The “3 Cs” model for vaccine hesitancy.**

| **“3 Cs” of vaccine hesitancy** | **Components** |
| --- | --- |
| **Confidence** | - Trust in safety and efficacy of vaccines - Trust in the system that delivers vaccines, including reliability and competence of health services and professionals. - Trust in the motivations of policy-makers who decide on the needed vaccines |
| **Complacency** | - Low perceived risk of vaccine-preventable diseases - Vaccination not deemed a necessary preventive action - Conflicting priorities seen to be more important at that point in time - Risk/benefit - Self-efficacy, or the perceived or real ability of a person to take action |
| **Convenience** | - Physical availability *of vaccines and vaccination services* - Affordability - Geographic accessibility *of vaccination services* - Language and literacy affecting ability to understand - Appeal of immunization services - Quality of services, i.e., the degree to which vaccination services are delivered at a time and place that is convenient and comfortable |

* Italicized items were added by the authors to the original model for clarity and relevance to this study.

**Supplementary Figure 1: Sankey diagram describing vaccination coverage and conditional timeliness among 134 children in their first year of life**

**
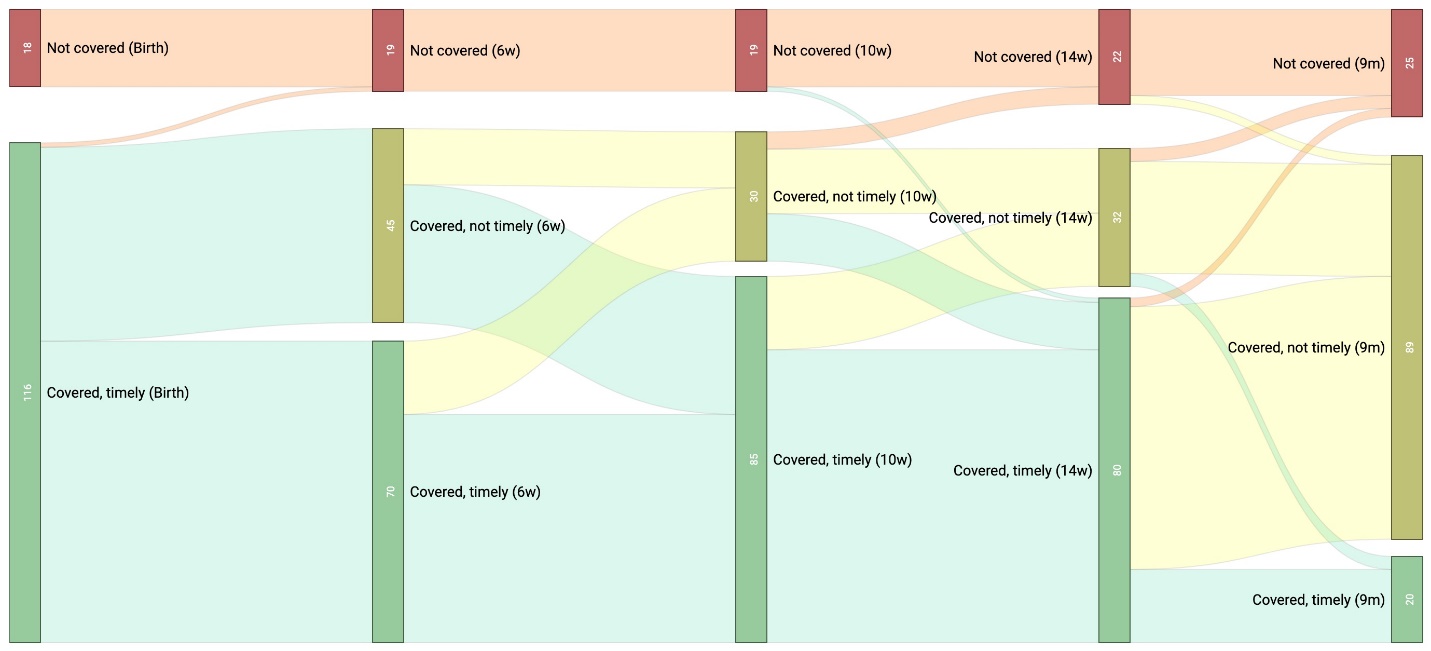
**

Vertical bars show the numbers of children with the corresponding vaccination status. Green represents children who received timely vaccinations; yellow represents children with delayed vaccinations; red represents unvaccinated children.

**Cross Sectional Survey Questionnaire**

**Start of Block: A. Mother Demographics**

Q1 Participant ID 
*Format:  0-0-0-00-A*

________________________________________________________________

Q2 Study phase

- 1 - Practice (1)
- 2 - Training (2)
- 3 - Cross-sectional survey (3)
- 4 - Longitudinal study (4)

Q3 Location

- Urban (1)
- Rural (2)

Q4 Client type

- Index (enrolled from health facility) (1)
- Referral (enrolled from the community) (2)

Q5 *Interviewer: Please remember to turn your phone to silent.*

Q6

How old are you?

________________________________________________________________

Q7 What is your marital status?

- Married (1)
- Widowed (2)
- Divorced / Separated (3)
- Never married (4)

Q8 Which of the following best describes your current employment status?
*[Interviewer: read responses aloud]*

- Student (1)
- Unemployed / Housewife (2)
- Self-employed (3)
- Casual laborer (4)
- Employed (5)
- Other (specify): (6) ________________________________________________

Q9 What is the highest level of education you have completed?

- None (1)
- Standard 1-6 (2)
- Standard 7 (3)
- Form 1-4 (4)
- Form 5 or higher (5)

Q10 How many women do you know in your street / village / neighborhood who are pregnant in their last trimester?

- 0 (1)
- 1 (2)
- 2 (3)
- 3+ (4)
- Other (specify): (5) ________________________________________________

Q11 I would like you to read this sentence to me.
*[Interviewer: show the sentence below to the respondent.]*

 **Parents must care for their children.**

 *[If respondent cannot read whole sentence, ask: "Can you read part of the sentence to me?"]*

- Cannot read at all (1)
- Able to read only parts of sentence (2)
- Able to read whole sentence (3)
- Unable because of physical / cognitive impairment (4)

*Skip To: End of Block If read = Cannot read at all*

Q12 Now I would like you to write this sentence down:

**"Farming is hard work."**


*[If respondent cannot write whole sentence, probe: "Can you write part of the sentence down?"]*

- Cannot write at all (1)
- Able to write only parts of sentence (2)
- Able to write whole sentence (3)
- Unable because of physical / cognitive impairment (4)

**End of Block: A. Mother Demographics**

**Start of Block: B. Reproductive History**

Q13 How many children have you given birth to?

________________________________________________________________

Q14 What was your age in years during your first pregnancy?

________________________________________________________________

Q15 What is the name of your youngest child (ages 12-23 months)?

- Name: (1) ________________________________________________

Q16 How old is ${Q15/ChoiceTextEntryValue/1}?

- Years: (1) ________________________________________________
- Months: (2) ________________________________________________

Q17 Is ${Q15/ChoiceTextEntryValue/1} a boy or a girl?

- Boy (1)
- Girl (2)

Q18 Where was ${Q15/ChoiceTextEntryValue/1} born?

- Home (1)
- Hospital (specify name): (2) ________________________________________________
- Health center (specify name): (3) ________________________________________________
- Public dispensary or clinic (specify name): (4) ________________________________________________
- Private clinic (specify name): (10) ________________________________________________
- Other (specify): (5) ________________________________________________

Q19 How many times did you receive antenatal care when you were pregnant with ${Q15/ChoiceTextEntryValue/1}?

- 0 (1)
- 1 (2)
- 2 (3)
- 3+ (4)

**End of Block: B. Reproductive History**

**Start of Block: C. Vaccination Knowledge**

Q20 What is vaccination?
*[Interviewer: Do not read the response aloud.]*

- When a child receives an injection or drops in the mouth for preventing diseases (1)
- Other (specify): (2) ________________________________________________
- Don't know (3)

*Display This Question:*

*If What is vaccination? [Interviewer: Do not read the response aloud.] = Other (specify):*

*Or What is vaccination? [Interviewer: Do not read the response aloud.] = Don't know*

Q21 *INTERVIEWER: Tell respondent that vaccinations are when a child receives an injection or drops in the mouth for preventing diseases.*

Q22 Can you name some diseases that can be prevented in young babies by vaccinations?

*[Interviewer: Do not read the responses aloud.]*
*[Probe until parent cannot name any more diseases. Mark all that apply.]*

- Tuberculosis (1)
- Influenza (2)
- Hepatitis B (3)
- Polio (4)
- Tetanus (5)
- Diphtheria (6)
- Pertussis (7)
- Measles (8)
- Mumps (9)
- Rubella (10)
- Meningococcal disease (11)
- Pneumococcal disease (12)
- Diarrhea (rotavirus) (13)
- Malaria (14)
- Other (specify): (15) ________________________________________________
- ⊗Cannot name any diseases (16)

Q23
Can you name some common side effects of getting vaccinated?
*[Interviewer: Do not read the responses aloud.]*
*[Probe until parent cannot name any more side effect. Mark all that apply.]*

- Injection site reactions (pain, swelling, and redness) (1)
- Mild fever (2)
- Shivering (3)
- Fatigue / Sleepiness (4)
- Fussiness or Crying (5)
- Other (specify): (6) ________________________________________________
- ⊗Cannot name any side effects (7)

**End of Block: C. Vaccination Knowledge**

**Start of Block: D. Vaccination Attitudes**

Q24 How confident are you in the effectiveness of vaccines in protecting your child from diseases?

- Very confident (1)
- Somewhat confident (2)
- Not confident (3)

Q25 How confident are you that the vaccines your child receives are safe?

- Very confident (1)
- Somewhat confident (2)
- Not confident (3)

Q26 How important is it to you that your baby receives all the vaccines recommended for him / her?

- Very important (1)
- Somewhat important (2)
- Not important (3)

Q27 How important is it to you that your baby receives vaccines according to the recommended schedule?

- Very important (1)
- Somewhat important (2)
- Not important (3)

Q28 How important do you think vaccines are for keeping your baby healthy?

- Very important (1)
- Somewhat important (2)
- Not important (3)

Q29 Now I will read you some statements.  Please tell me if you agree or disagree with them.

|  | Agree (1) | Disagree (2) | Not sure (3) |
| --- | --- | --- | --- |
| Vaccinations can prevent deadly diseases. (1) |  |  |  |
| One dose of vaccine is enough. Multiple doses are not required. (4) |  |  |  |
| It is safe for a child to receive multiple vaccines in one appointment. (3) |  |  |  |
| My child does not need vaccines for diseases that are not common anymore. (2) |  |  |  |
| Children who are healthy do not need vaccinations (9) |  |  |  |
| Some babies are too small to get vaccines -- it is better to wait until they are older. (10) |  |  |  |
| I am concerned about the side effects of vaccines. (6) |  |  |  |
| The benefits of vaccines outweigh the minor side effects like minor fever, fussiness, or soreness at the site of the injection. (8) |  |  |  |
| Vaccines work only if given before the disease strikes (7) |  |  |  |
| Having my child vaccinated is important for the health of others in my community. (5) |  |  |  |

Q30 Who or what is your main source of information about childhood vaccinations?
(Mark only the main source)

- Health facility / health worker (1)
- Friend or Family member (2)
- Village leader (3)
- Religious leaders (4)
- School (5)
- Media (6)
- Campaign / megaphones (7)
- Cell phone messages (8)
- Others (specify): (9) ________________________________________________
- No source (10)
- Don't know (11)

**End of Block: D. Vaccination Attitudes**

**Start of Block: E. Vaccination Practices**

Q31 We would like to know who among those living in your household have ever received any vaccine. This includes you and your child, and any vaccinations received in a campaign or on an immunization day.
(Write DK if respondent does not know)

|  | Total number of household members in this group (1) | Total number in this group ever vaccinated (2) |
| --- | --- | --- |
| Mother (You) (1) |  |  |
| Other adults ≥ 18 years of age (2) |  |  |
| Children 0-4 years of age (3) |  |  |
| Children 5-9 years of age (4) |  |  |
| Children 10-17 years of age (5) |  |  |

Q32 In total, including you, how many adults and children live in your household

- Adults >=18 years of age (1) ________________________________________________
- Children 0-17 years of age (2) ________________________________________________

Q33 Where do you and others in your household usually go to receive vaccines?
(Mark all that apply)

- ${Q18/ChoiceGroup/SelectedChoicesTextEntry} (1)
- Hospital (2)
- Health center (3)
- Public dispensary or clinic (4)
- Private clinic (5)
- Health care worker visits to house / community / village (6)
- Other (specify): (7) ________________________________________________
- ⊗Don't know (8)

Q34 How many times did you receive tetanus toxoid vaccinations when you were pregnant with ${Q15/ChoiceTextEntryValue/1}?

- 0 (1)
- 1 (2)
- 2 (3)
- 3+ (4)
- Don't know (5)

Q35 Do you have a copy of the tetanus toxoid vaccination card?
*[Interviewer: Show example of the tetanus toxoid vaccination card to the respondent.]*

- Yes (1)
- No (2)

*Skip To: Q39 If Do you have a copy of the tetanus toxoid vaccination card? [Interviewer: Show example of the teta... = No*

Q36 [INTERVIEWER] Please enter the dates for the Tetanus Toxoid vaccine (dd/mm/yyyy)
Enter "44" if the card shows that a dose was given, but no specific date is recorded.

Q37 **Tetanus Toxoid**

- Vaccination 1 (1) ________________________________________________
- Vaccination 2 (2) ________________________________________________
- Vaccination 3 (3) ________________________________________________
- Vaccination 4 (4) ________________________________________________
- Vaccination 5 (5) ________________________________________________

Q38 [INTERVIEWER] Take a picture of the card.

Q39 Have any of the following individuals ever taken ${Q15/ChoiceTextEntryValue/1} for vaccinations? (Mark all that apply, read aloud.)

- Mother (the respondent) (1)
- Father (2)
- Grandmother (3)
- Grandfather (4)
- Other relative (5)
- Friend (6)
- Other (specify): (7) ________________________________________________
- ⊗No-one (8)

Q40 Did you receive a vaccination card for ${Q15/ChoiceTextEntryValue/1}?

- Yes (1)
- No (2)
- Don't know (3)

*Skip To: Q55 If Did you receive a vaccination card for ${q://QID122/ChoiceTextEntryValue/1}? = No*

*Skip To: Q55 If Did you receive a vaccination card for ${q://QID122/ChoiceTextEntryValue/1}? = Don't know*

Q41 INTERVIEWER: Please ask the mother to show you the vaccination card or other document and record the dates of vaccination (dd/mm/yyyy).  Enter "44" if the card shows that a dose was given, but no specific date is recorded.

Q42 **BCG Vaccine**(Tuberculosis)
*Needle, right shoulder*

- At birth or the first clinic visit (1) ________________________________________________
- Scar is present (1) (2)
- Scar is present (2) (3)
- If no scar, re-do at 3 months (4) ________________________________________________

Q43 INTERVIEWER: For the next five questions, please ask the mother to show you the vaccination card or other document and record the dates of vaccination (dd/mm/yyyy).  Enter "44" if the card shows that a dose was given, but no specific date is recorded.

| Vaccine | 0 (Birth) | 1 (6 Weeks) | 2 (10 Weeks) | 3 (14 Weeks) |
| --- | --- | --- | --- | --- |
| Q45 **Polio - OPV**  *Drops, oral* |  |  |  |  |
|  |  |  |  |  |
| Q46 **Polio - IPV**  *Needle, right thigh* |  |  |  |  |
|  |  |  |  |  |
| Q47 **DTP - Hep B - HIb Pentavalent**  (Diphtheria, Tetanus, Pertussis, Hepatitis B, and Haemophilus Influenza b)  *Needle, left thigh* |  |  |  |  |
|  |  |  |  |  |
| Q48 **PCV13** (Pneumococcal disease)  *Needle, right thigh* |  |  |  |  |
|  |  |  |  |  |
| Q49 **Rotarix**(Diarrhea / Rotavirus)  *Drops, oral* |  |  |  |  |

Q50 INTERVIEWER: Please ask the mother to show you the vaccination card or other document and record the dates of vaccination (dd/mm/yyyy).  Enter "44" if the card shows that a dose was given, but no specific date is recorded.

| Vaccine | 1. Months (1) | 18 Months (2) |
| --- | --- | --- |
| Q52 **MR**  (Measles and Rubella)  *Needle, left shoulder* |  |  |

Q53 Vitamin A Supplements & Worm Medication
[INTERVIEWER] Please place a checkmark for the month given

|  | 6 Months (1) | 12 Months (2) | 18 Months (3) | 24 Months (4) |
| --- | --- | --- | --- | --- |
| **Vitamin A** *Drops, oral* (1) |  |  |  |  |
| **Worm Medication** *Tablets, oral* (2) |  |  |  |  |

Q54 [INTERVIEWER] Click below to take a picture of the card.

Q55 Did you ever know ${Q15/ChoiceTextEntryValue/1} was supposed to get a vaccine dose, but for some reason, did not get it?

- Yes (1)
- No (2)
- Don't know (3)

*Skip To: End of Block If Did you ever know ${q://QID122/ChoiceTextEntryValue/1} was supposed to get a vaccine dose, but fo... = No*

*Skip To: End of Block If Did you ever know ${q://QID122/ChoiceTextEntryValue/1} was supposed to get a vaccine dose, but fo... = Don't know*

Q56 What were the reasons for not vaccinating ${Q15/ChoiceTextEntryValue/1}?
(**Do not read aloud.** Mark all that apply.)

- Did not think it was needed (1)
- Did not think it was effective (2)
- Side effects (3)
- Bad experience with previous vaccination (4)
- Family member or friend advised against it (5)
- Fear of injection (6)
- Busy (7)
- No transportation (8)
- Child was sick (9)
- Vaccine not available (10)
- Vaccinator busy / not available (11)
- Long waiting times (12)
- Asked to return at another time (13)
- Unfriendly vaccination staff (14)
- Other (specify): (15) ________________________________________________
- ⊗No specific reason for not vaccinating (16)

*Display This Question:*

*If If What were the reasons for not vaccinating ${q://QID122/ChoiceTextEntryValue/1}? (Do not read alou... q://QID112/SelectedChoicesCount Is Greater Than 1*

*Carry Forward Selected Choices - Entered Text from "What were the reasons for not vaccinating ${q://QID122/ChoiceTextEntryValue/1}? (Do not read aloud. Mark all that apply.)"*

Q57 What was the **primary** reason for not vaccinating ${Q15/ChoiceTextEntryValue/1}?
(Mark one)

- Did not think it was needed (1)
- Did not think it was effective (2)
- Side effects (3)
- Bad experience with previous vaccination (4)
- Family member or friend advised against it (5)
- Fear of injection (6)
- Busy (7)
- No transportation (8)
- Child was sick (9)
- Vaccine not available (10)
- Vaccinator busy / not available (11)
- Long waiting times (12)
- Asked to return at another time (13)
- Unfriendly vaccination staff (14)
- Other (specify): (15)
- ⊗No specific reason for not vaccinating (16)

**End of Block: E. Vaccination Practices**

**Start of Block: F. Household Decision Making**

Q58 Who in your family has the final say in the decision to seek medical care for a sick child including the cost of that care (medicine, transport, etc)?

- I as the mother (1)
- My partner and I (2)
- My partner (3)
- Grandparent of child (4)
- Someone else (5)

Q59 Who in your family has the final say in the decision to make large household purchases?

- I as the mother (1)
- My partner and I (2)
- My partner (3)
- Grandparent of child (4)
- Someone else (5)

Q60 Who in your family has the final say in the decision to visit relatives or friends?

- I as the mother (1)
- My partner and I (2)
- My partner (3)
- Grandparent of child (4)
- Someone else (5)

**End of Block: F. Household Decision Making**

**Start of Block: G. SES**

Q61 Type of residence 
(Mark one)

- Room(s) (1)
- House (2)
- Apartment (3)
- Other (4)

Q62 Main material of the floor 
(Mark one)

- Earth (1)
- Cement (2)
- Other (3)

Q63 How many rooms are used for sleeping?
_____________________________________________________

Q64 Does your household own...
(Mark all that apply, read one-by-one)

- Electricity (1)
- A radio (2)
- A television (3)
- A mobile phone (4)
- An iron (5)
- A refrigerator (6)
- A motorcycle or motor scooter (7)
- A car or truck (8)
- A bank account (9)
- Tap water in the house (10)
- None of the above (11)

Q65 What kind of toilet facility do members of your household usually use?
(Mark one, read responses aloud)

- Flush toilet (1)
- Pit latrine (2)
- Other (3)

Q66 What is the tenure status of the main residence?
(Mark one)

- Owner occupied (1)
- Employer occupied (2)
- Rented (3)
- Other (4)

Q67 Do you watch television?
(Mark one, read responses aloud)

- Almost every day (1)
- At least once a week (2)
- Less than once a week (3)
- Not at all (4)

Q68 Are you currently covered by any health insurance?

- Yes (1)
- No (2)

Q69 How long does it take you to walk to the ...
*(Record answer in minutes)*

- Nearest dispensary (1) ________________________________________________
- Nearest health center (2) ________________________________________________

**End of Block: G. SES**

**Start of Block: H. Mobile Phone Ownership and Use**

Q70 How often do you use a mobile phone?

- Every day (1)
- At least once a week (2)
- Less than once a week (3)
- Never (4)

*Skip To: End of Block If mpuse = Never*

Q71 Which of the following mobile networks do you connect to with the mobile phone you use? 
(Mark all that apply)

- Airtel (1)
- Sasatel (2)
- Tigo (3)
- TTCL (4)
- Other (specify): (9) ________________________________________________
- Vodacom (5)
- ZanTel (6)
- Halotel (7)
- Smile (8)
- ⊗Don't know (10)

Q72 Who owns the mobile phone that you use?

- Self (1)
- Spouse (Husband / Wife) (2)
- You and your spouse jointly (3)
- Other household member (specify): (4) ________________________________________________
- Friend (5)
- Other (specify): (6) ________________________________________________

Q73 Does the mobile phone you use...

|  | Yes (1) | No (2) | Don't know (3) |
| --- | --- | --- | --- |
| Access internet? (3) |  |  |  |
| Have a touchscreen? (4) |  |  |  |

Q74 Have you ever used the mobile phone you use to:
(Select all that apply)

- Make phone calls (1)
- Receive phone calls (2)
- Send SMS (3)
- Open and read SMS (4)
- Receive money (5)
- Send money (6)
- Browse the internet (7)
- Play games (8)
- Use Facebook (9)
- ⊗None of the above (10)

Q75 If an important message were to be delivered to you on the mobile phone you use, how soon would you be able to receive it?

- Same day (1)
- Next day (2)
- 2-3 days (3)
- 4-7 days (4)
- >7 days (5)

Q76 In the past month, how often have you had problems with...

|  | Less than once per week (1) | One or more times per week (2) | Most days (3) |
| --- | --- | --- | --- |
| Charging phone (1) |  |  |  |
| Connection problems (2) |  |  |  |

Q77 How much do you spend per week on phone charges?

- 0 - 499 TSH (1)
- 500 - 999 TSH (2)
- 1,000 - 1,999 TSH (3)
- 2,000 - 4,999 TSH (4)
- 5,000 - 9,999 TSH (5)
- 10,000+ TSH (6)

Q78 Do you use any bundles?

- Daily (1)
- Weekly (2)
- Monthly (3)
- ⊗None (4)

Q79 How many times has a mobile phone you use been lost or stolen?

- Times lost: (1) ________________________________________________
- Times stolen: (2) ________________________________________________

**End of Block: H. Mobile Phone Ownership and Use**

**Start of Block: I. Mobile Phone Use for Vaccinations**

*Display This Question:*

*If mpuse != Never*

Q80 I will read you some statements. Please tell me if you would find these very useful, somewhat useful, or not useful.

How useful would it be to use your mobile phone for...

|  | Very useful (1) | Somewhat useful (2) | Not useful (3) |
| --- | --- | --- | --- |
| Receiving reminders about your child's upcoming vaccination appointments? (1) |  |  |  |
| Receiving information about vaccination availability and stockouts? (2) |  |  |  |
| Receiving information about vaccination clinic opening hours and closures (3) |  |  |  |
| Receiving tips and information on how to keep your child healthy (4) |  |  |  |

*Display This Question:*

*If mpuse != Never*

Q81 Would you like to receive reminders about your child's upcoming vaccinations on a mobile phone?

- Yes (1)
- No (2)
- Unsure / Maybe (3)

*Skip To: End of Block If mpreminders = No*

*Display This Question:*

*If mpuse != Never*

Q82 How would you prefer to receive the reminder?

- SMS (1)
- Phone call (2)
- No preference (3)

*Display This Question:*

*If mpuse != Never*

Q83 When would you prefer to receive the reminder?
(Mark all that apply)

- One week before the due date (1)
- One day before the due date (2)
- On the due date (3)
- No preference (4)

*Display This Question:*

*If mpuse != Never*

Q84 At what time would you prefer to receive the reminder?
(Mark all that apply)

- Mornings (1)
- Mid-day (2)
- Evenings (3)
- No preference (4)

**End of Block: I. Mobile Phone Use for Vaccinations**

**Start of Block: J. WTA/WTP**

Q85 Children should be vaccinated at birth, and at the ages of 4 weeks, 8 weeks, 12 weeks, 8 months, and 18 months.  Currently vaccinations are provided free of charge in Tanzania.
Imagine you have another child.
Do you expect to get the child vaccinated at each of these times?

- Yes (1)
- No (2)

*Skip To: Q87 If Children should be vaccinated at birth, and at the ages of 4 weeks, 8 weeks, 12 weeks, 8 months,... = No*

Q86 Now consider the possibility that the government of Tanzania no longer provides free vaccinations, and that there is a cost for each vaccination for your child.  If you were asked to pay TSH 1000 each time your child receives a vaccination, would you get your child vaccinated?

- Yes (1)
- No (2)

*Skip To: Q90 If Now consider the possibility that the government of Tanzania no longer provides free vaccinations... = Yes*

*Skip To: Q88 If Now consider the possibility that the government of Tanzania no longer provides free vaccinations... = No*

Q87 Now consider the possibility that the government provides a subsidy to people who ensure that their children receive timely vaccinations—which means coming to the clinic on a specifically scheduled date and your child is a specific age.  If you were offered TSH 1000 each time your child receives a vaccination on time, would you get your child vaccinated?

- Yes (1)
- No (2)

*Skip To: Q89 If Now consider the possibility that the government provides a subsidy to people who ensure that the... = Yes*

*Skip To: Q91 If Now consider the possibility that the government provides a subsidy to people who ensure that the... = No*

Q88 If you were asked to pay TSH 500 each time your child receives a vaccination, would you get your child vaccinated?

- Yes (1)
- No (2)

*Skip To: Q92 If If you were asked to pay TSH 500 each time your child receives a vaccination, would you get your... = No*

Q89 If you were offered TSH 500 each time your child receives a vaccination on time, would you get your child vaccinated?

- Yes (1)
- No (2)

Q90 If you were asked to pay TSH 2000 each time your child receives a vaccination, would you get your child vaccinated?

- Yes (1)
- No (2)

*Skip To: Q92 If If you were asked to pay TSH 2000 each time your child receives a vaccination, would you get your... = Yes*

Q91 If you were offered TSH 2000 each time your child receives a vaccination on time, would you get your child vaccinated?

- Yes (1)
- No (2)

*Skip To: Q93 If If you were offered TSH 2000 each time your child receives a vaccination on time, would you get y... = No*

Q92 What would be the maximum amount you would be willing to pay for each of your child’s vaccinations?
(Enter the amount in TSH)

________________________________________________________________

*Display This Question:*

*If Now consider the possibility that the government provides a subsidy to people who ensure that the... = No*

Q93 How much money would one need to offer you in order for you to get each of your child’s vaccinations on time?
(Enter the amount in TSH)

________________________________________________________________

Q94 If you were offered the following rewards in exchange for getting your child vaccinated on time, how would you rank the following?  


Please rank rewards from 1 to 6, where 1 = First choice and 6 = Last choice.

| Put rewards in order |
| --- |
| ______ Reimbursement of transport cost (1) |
| ______ Pharmacy voucher for TSH 2,000 (3) |
| ______ Free health check for mother (4) |
| ______ Birth certificate (5) |
| ______ Phone credit of TSH 2,000 (6) |
| ______ Lottery ticket to win TSH 10,000 (7) |
| ______ Lottery ticket to win TSH 20,000 (8) |

**End of Block: J. WTA/WTP**

Q162 Notes

________________________________________________________________

________________________________________________________________

________________________________________________________________

________________________________________________________________

________________________________________________________________

**MOTHERS FOCUS GROUP GUIDE**

**A. Focus group participant demographic survey**

***[Complete the survey as you are waiting for the mothers to arrive]***

1. Focus group date
2. Focus group participant ID (e.g., M001, M002 etc.)
3. What is your age (in years)?
4. What is the highest level of education you have completed?
   1. None
   2. Primary or less (grades 1-7)
   3. Secondary ( Form 1-to Form 4)
   4. Secondary (Form 5-6)
   5. Post-secondary
5. How many children have you given birth to?
6. Did your youngest child ever receive any vaccinations to prevent getting diseases? This includes vaccinations received during well child visits, in campaigns, immunization days or child health days?
   1. Yes
   2. No
7. Were you ever late for any vaccinations for any of your children?
   1. Yes
   2. No
8. Did you ever miss any vaccinations for any of your children?
   1. Yes
   2. No
9. Do you use a mobile phone?
10. Yes
11. No – ***STOP.***
12. What type of mobile phone do you use? ***[Show card with phone images to help choose]***
13. Basic phone
14. Feature phone
15. Blackberry/Smart phone/touch screen phone
16. Have you ever received any health information or health services via your mobile phone?
17. Yes
18. No

13. How much money do you spend on average on mobile phone credit every week?

1. <500 TSH
2. 500-1000 TSH
3. 1001-2000 TSH
4. 2001-3000 TSH
5. 3001-4000 TSH
6. 4001-5000 TSH
7. >5000 TSH

**MOTHERS FOCUS GROUP GUIDE**

**Focus group discussion**

[Settle in time – 10 min]

Thank you for joining us today. Before we begin the discussion, we first we need to review our research consent form.

[Overview of consent form – 5 min]

Introduction

We are here today to discuss the topic of childhood vaccinations. What you all have in common is that you are mothers and therefore face the decision of whether or not to vaccinate your children.

The purpose of this focus group is to learn about when and how often parents choose to vaccinate their child. It is not to collect information about your health or your child’s health. I hope you will feel free to discuss your personal opinions, but with an intention towards helping us understand vaccination choices.

There is no right or wrong answer today. Everyone here has an important perspective to share and you all have expertise on this subject. I would like to hear as many different ideas as possible today, so I may ask you to be brief to give others a chance to share. I hope you will understand and be OK with this request.

Finally, we ask that each of you respect the privacy of your fellow focus group participants by keeping the comments made today confidential and by not sharing them with anyone outside this group. Any questions?

*[Discussion – 1.5 hours]*

Let us begin:

| **0. ICE BREAKER** |
| --- |
| ***[Show the printout of the word “VACCINATION” and ask participants to tell you what comes to mind.]*** |

| **I. HOUSEHOLD DECISION-MAKING** |
| --- |
| *I would like to understand how decisions about vaccinations are made in your family.*  1.1 Can you tell me how decisions about your child’s health are made in the family?  *PROBES:*   - *Who makes the final decision about whether your child receives health services such as vaccinations?* - *(For women who are not the primary decision makers) What is your role in the decision-making process?* - *Who else do you seek advice from about your child’s health?*   1.2 What are some factors that influence your family’s decision to vaccinate your child?  *PROBES:*   - *How often do you discuss your child’s vaccinations with your spouse?* - *What other things about the well child visit do you discuss?* - *How do you choose when and where to vaccinate your child?* |

| **II. BARRIERS TO VACCINATIONS** |
| --- |
| *Now I would like to ask you about any challenges you may face in trying to vaccinate your child. I want to start by asking how many of you vaccinate your child. Have you ever missed vaccinating your child? Have you ever experienced delays or difficulties vaccinating your child?*  2.1 What are some things that make it hard to vaccinate your child?  ***[Record reasons on the left side of the board]***  ***[Wait sufficient time for the participants to respond before probing.]***  *PROBES:*   - *Do you need permission from your spouse or other family members to bring your child to the vaccination clinic? Is this ever a problem?* - *Does having to bring your child to the vaccination clinic ever conflict with your job or other daily responsibilities?* - *Have you ever had trouble getting to the vaccination clinic? In what ways?* - *Is the cost of vaccinating/taking your child to the vaccination clinic a barrier?* - *Have you ever had a bad experience trying to vaccinate your child?* - *Have you ever come to the vaccination clinic but been denied vaccinations? What was the reason?* - *Can you think of any other challenges you may have faced in vaccinating your child?*   *For those of you who missed vaccinating your child:*  2.2 What were some reasons for missing the appointment?  *For those of you who do not vaccinate your child:*  2.3 Why do you not vaccinate your child?  *PROBES:*   - *Have you ever tried to vaccinate your child? What happened?* - *Compared with your older children, how did your decisions differ for your youngest child?*   2.4 What would need to change for you to start vaccinating your child? |

| **III. INCENTIVES** |
| --- |
| *A goal of this project is to help reduce barriers to vaccinations, so I would like to ask you for your input on things that will motivate mothers like you to vaccinate your child.*  3.1 What are some things that will make it easier to vaccinate your child or motivate you to make a decision in favor of it?  ***[Record responses on the right side of the board]***  ***[Wait sufficient time for the participants to respond before probing.]***  *PROBES:*   - *What kinds of goods or services would motivate mothers to vaccinate their children?* - *How would getting cash for vaccinating children influence mothers’ decisions?* - *How large would that cash amount need to be?* - *How would a non-cash reward of the same value as the cash reward (like a bag of groceries, or airtime for you cell phone) influence mothers’ decision?* - *How does the non-cash reward compare to the cash incentive?*   3.2 What has been your experience with lotteries?  *PROBES:*   - *Would you prefer a lottery with a high reward but low chance of winning, or a lottery where the reward amount is low but the chance of winning is high?* - *Which would you prefer, and why: cash or some other prize?* - *What would the minimum prize value need to be for you to join the lottery?* - *After not winning in a lottery at the first try, would you play that lottery again?*   3.3 Are you or anyone in your family a part of the National Health Insurance scheme (~75,000 annual) or the Community Health Fund (~10,000 TSH annual) or any other voluntary insurance schemes?  *PROBES:*   - *Why not?* - *Is the contribution cost/sign-up fee a barrier?*   3.4 Have you received a birth certificate for any of your children? ***[Note count]***  *PROBES:*   - *Why not?* - *If you were to receive your child’s birth certificate as a reward for completing all of your child’s vaccinations, how would that affect your decision to vaccinate?* |

*[Closing question]*

Thank you for all your feedback today. Is there anything else you would like to share about your experience vaccinating your child/children?
